# Supplementary material for: Viral coinfection is shaped by host ecology and virus–virus interactions across diverse microbial taxa and environments
Source: Virus Evol. 2017 Apr 27;3(1):vex011. doi: 10.1093/ve/vex011 (PMC5407056; doi:10.1093/ve/vex011)

Supplementary Table 1. Description of data sets used in this study.

	Flores et al. 2011	Roux et al. 2015	Roux et al. 2014
Type of viral infection examined	lytic	all	all
Cultured viruses?	yes	no	no
Cultured hosts?	yes	yes	no
Infection detection method	lysis	sequence-based	sequence-based
Past viral infections?	no	yes	yes
Number of hosts tested (bacteria/archaea*)	1,005	5,492*	67
Number of viruses tested	499	12,498	106
Reference	(Flores *et al.*, 2011)	(Roux *et al.*, 2015)	(Roux *et al.*, 2014)

*Only the Roux et al. 2015 data set includes archaeal hosts.

Supplementary Figure 1. Diagnostic plots for ANOVA model using all variables on the proportion of tested phages infecting.

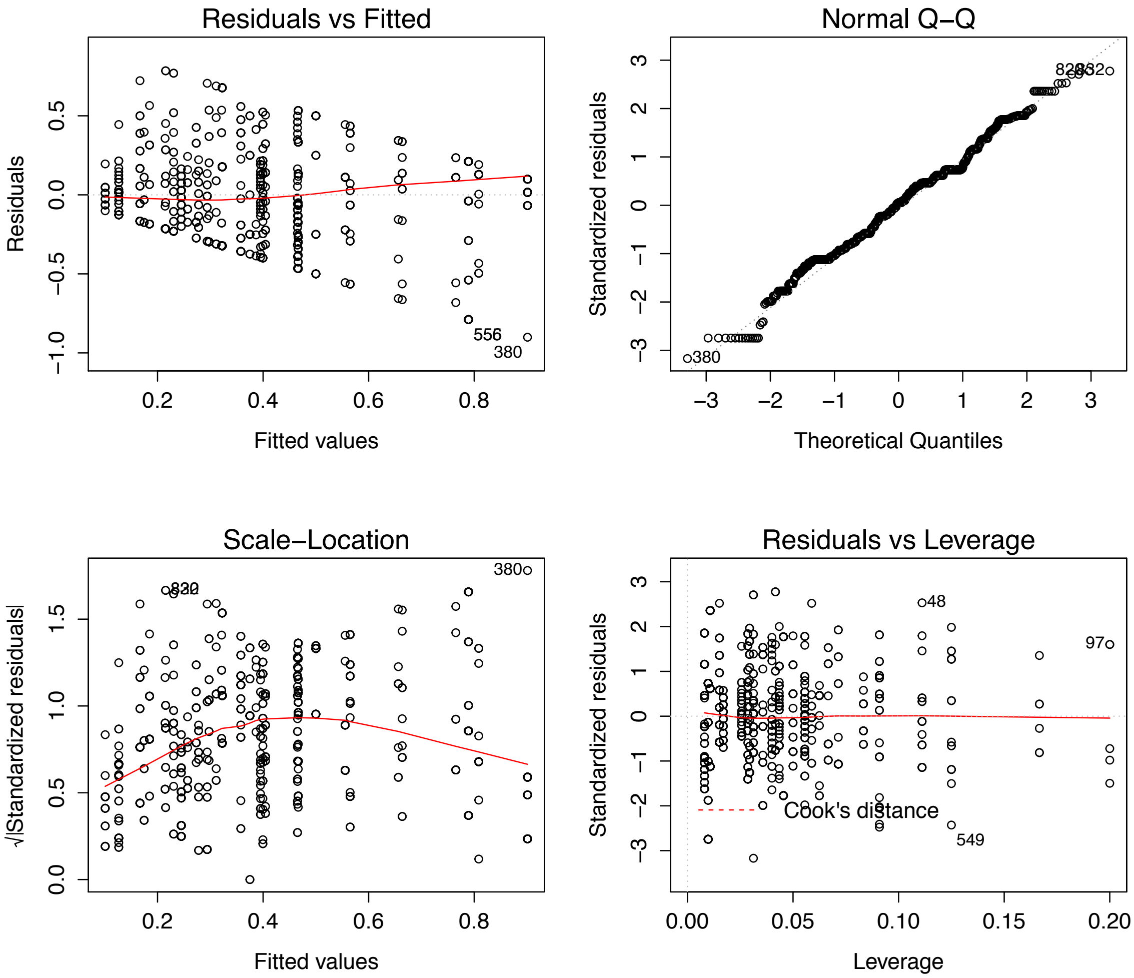

Supplementary Figure 2. Plots of all tested factors for the potential coinfection (cross-infectivity) data set. Potential coinfection is the number of phages that can infect a bacterial host, here measured as the proportion of tested phages infecting each host (represented by points). Point colors correspond to hosts in the same study. Note data points are offset by a random and small amount (jittered) to enhance visibility and reduce overplotting. All factors explaining a statistically significant proportion of the variation in the full model are depicted (A-E). Those factors selected after stepwise model selection using AIC are indicated with a blue outline around the plot. ANOVA tables for the full and reduced (blue outline) models are presented in F.

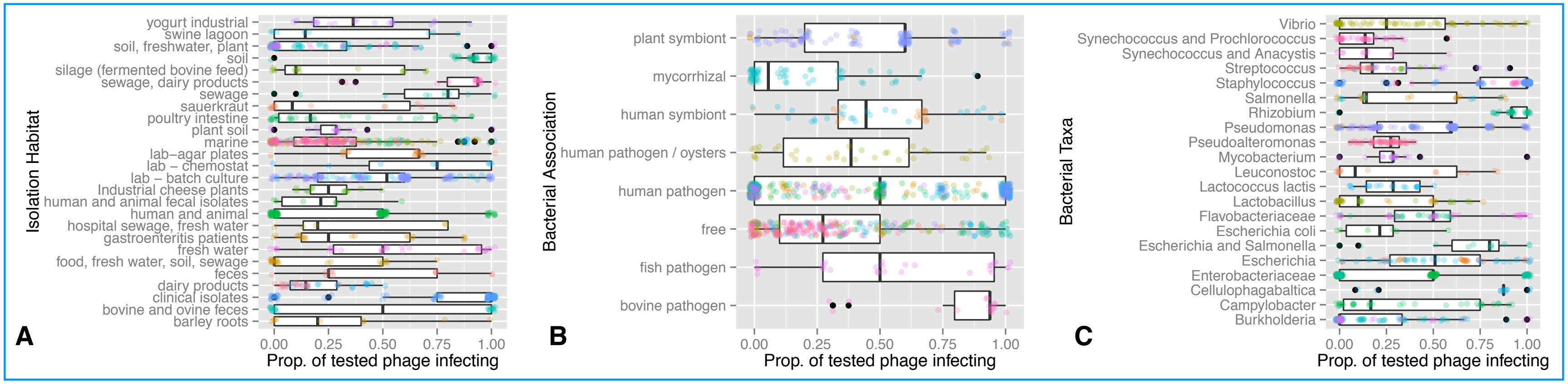

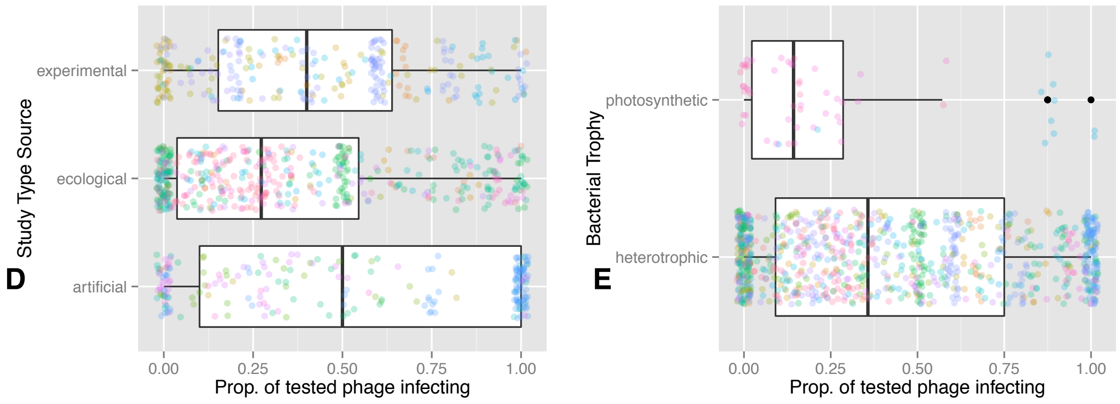

F

	Df	Sum Sq	Mean Sq	F value	Pr(>F)
Bacterialtrophy	1	1.6173	1.6173	19.3731	0
Majority.source	2	4.633	2.3165	27.7491	0
Bacterial.Association	7	8.4038	1.2005	14.3813	0
Isolation_Habitat_new	22	29.294	1.3315	15.9506	0
Bacteria_new	3	1.8345	0.6115	7.3251	1e−04
Residuals	969	80.8917	0.0835	NA	NA

	Df	Sum Sq	Mean Sq	F value	Pr(>F)
Bacterial.Association	7	10.6497	1.5214	18.2389	0
Isolation_Habitat_new	22	30.8534	1.4024	16.8127	0
Bacteria_new	5	4.259	0.8518	10.2115	0
Residuals	970	80.9121	0.0834	NA	NA

Supplementary Figure 3. Plots of all tested factors for culture coinfection. Plot A only includes genera with sample sizes >1 and non-zero mean extrachromocomal virus infection.

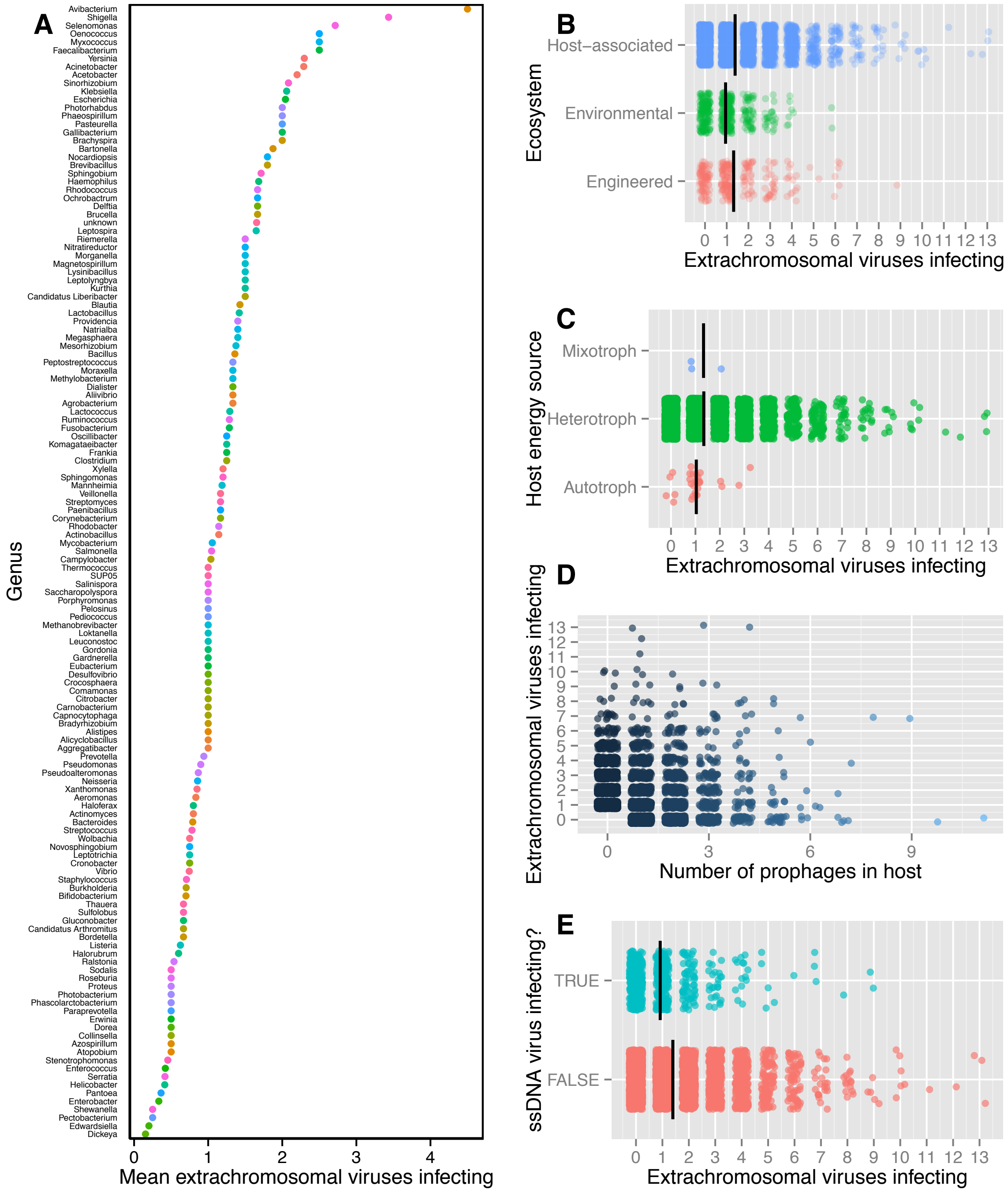

Supplementary Figure 4. Plots of all tested factors for single cell coinfection.

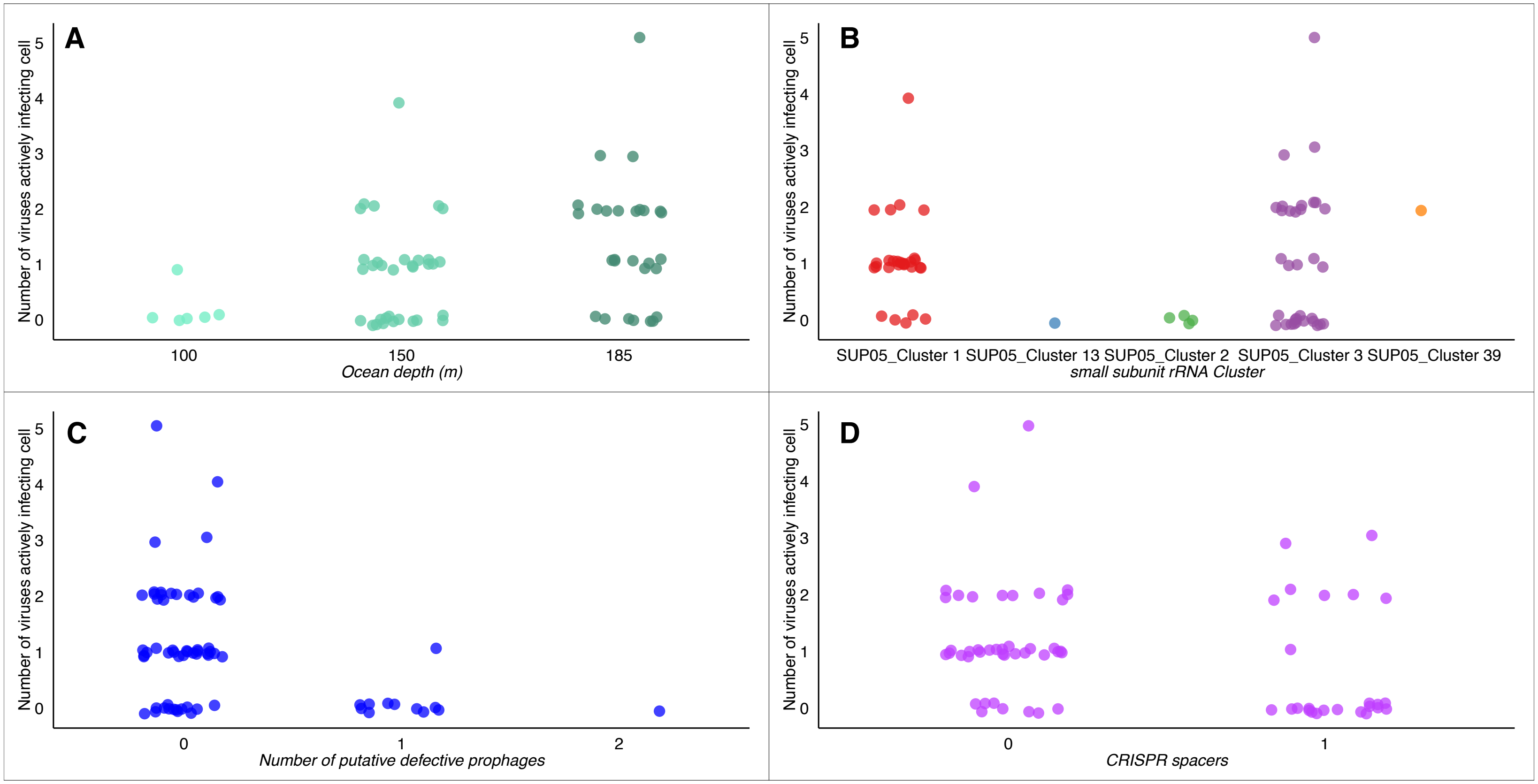

Supplementary Figure 5. Ecosystem of microbes influences potential and culture coinfection in similar ways. See Table S2 below for mean percentage difference.

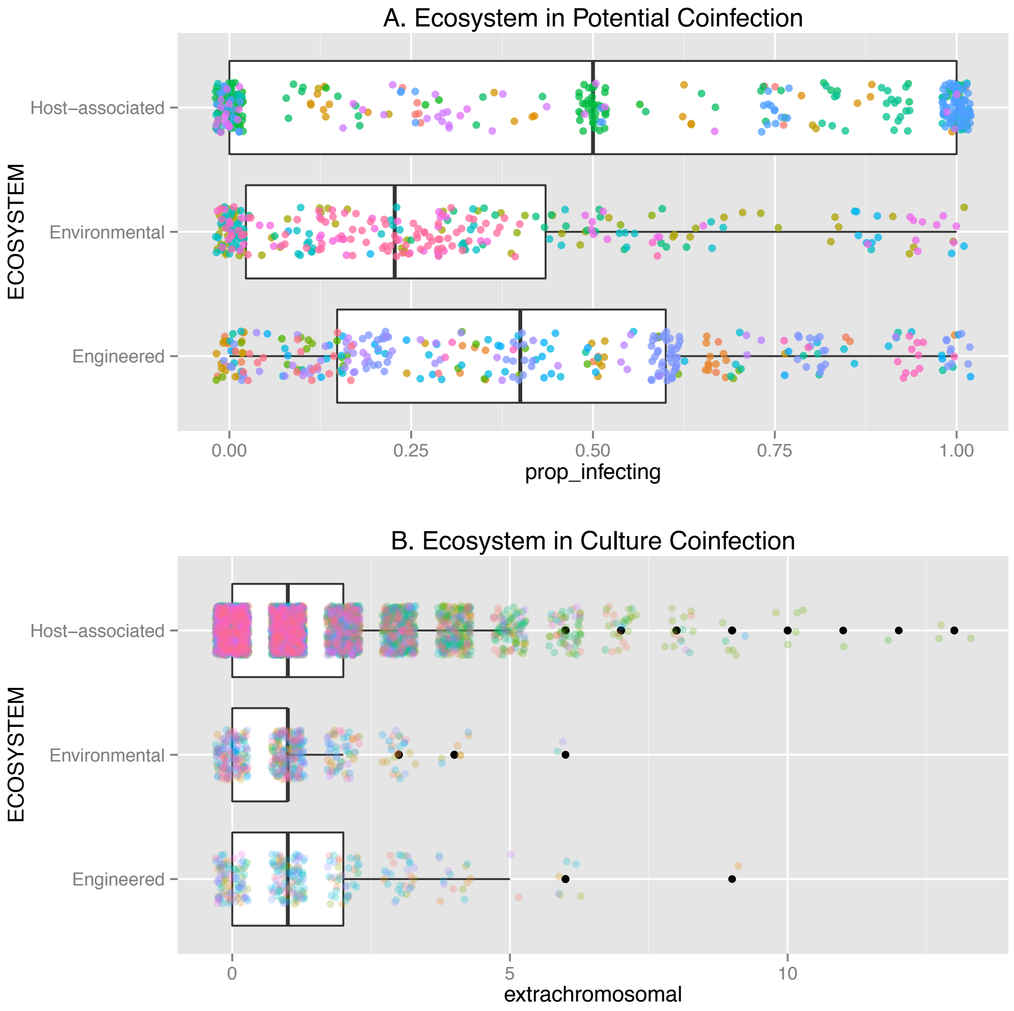

Supplementary Table 2. Percentage differences in potential and culture coinfection according to microbial ecosystem habitat are similar. Microbes isolated from host-associated ecosystems had the highest coinfection levels. The percentage difference of the other ecosystems relative to that host-associated baseline (i.e. 100%) is shown in each cell. See code in Figshare repository for calculations.

	Host-associated	Engineered	Environmental
**Potential Coinfection**	100%	82%	56%
**Culture Coinfection**	100%	95%	68%

Supplementary Figure 6. Heterotrophic hosts have higher average potential coinfection (cross-infectivity) and culture coinfection. The measures of coinfection are on different scales, but in percentage terms the difference was similar: Heterotrophic hosts had 58.59% higher cross-infectivity and 77.08% higher culture coinfection. See code in Figshare repository for calculations.

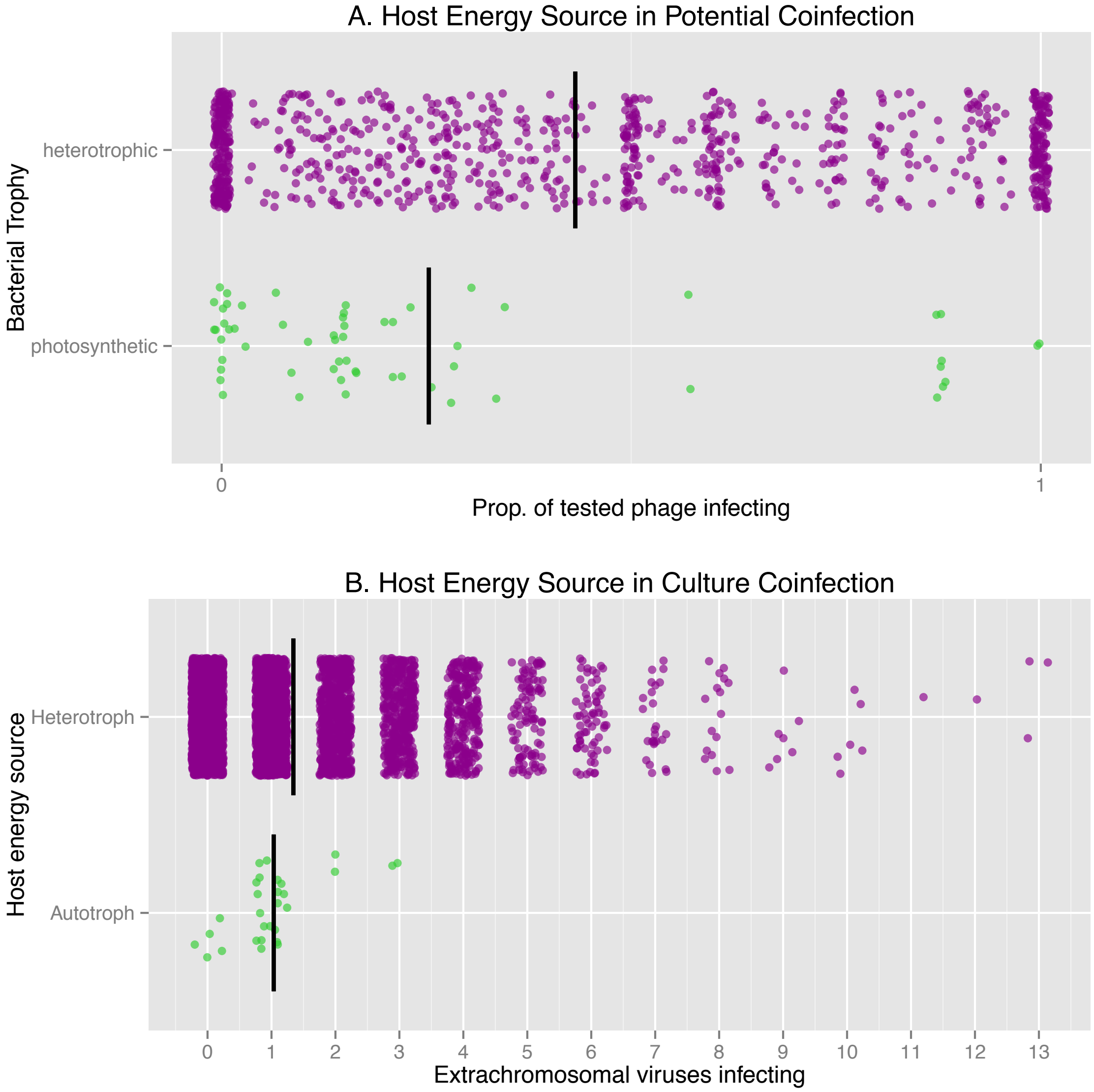

Supplement: Supplementary Data [file vex011_Supp.pdf]
